# The CsTIE1–CsAGL16 module regulates lateral branch outgrowth and drought tolerance in cucumber

**DOI:** 10.1093/hr/uhae279

**Published:** 2024-10-02

**Authors:** Jiacai Chen, Guangxin Chen, Jingyu Guo, Yuting He, Liu Liu, Shaoyun Wang, Chaoheng Gu, Lijie Han, Min Li, Weiyuan Song, Liming Wang, Xiaolan Zhang, Jianyu Zhao

**Affiliations:** Beijing Key Laboratory of Growth and Developmental Regulation for Protected Vegetable Crops, Department of Vegetable Sciences, China Agricultural University, Beijing 100193, China; Beijing Key Laboratory of Growth and Developmental Regulation for Protected Vegetable Crops, Department of Vegetable Sciences, China Agricultural University, Beijing 100193, China; Beijing Key Laboratory of Growth and Developmental Regulation for Protected Vegetable Crops, Department of Vegetable Sciences, China Agricultural University, Beijing 100193, China; Beijing Key Laboratory of Growth and Developmental Regulation for Protected Vegetable Crops, Department of Vegetable Sciences, China Agricultural University, Beijing 100193, China; Beijing Key Laboratory of Growth and Developmental Regulation for Protected Vegetable Crops, Department of Vegetable Sciences, China Agricultural University, Beijing 100193, China; Beijing Key Laboratory of Growth and Developmental Regulation for Protected Vegetable Crops, Department of Vegetable Sciences, China Agricultural University, Beijing 100193, China; Beijing Key Laboratory of Growth and Developmental Regulation for Protected Vegetable Crops, Department of Vegetable Sciences, China Agricultural University, Beijing 100193, China; Beijing Key Laboratory of Growth and Developmental Regulation for Protected Vegetable Crops, Department of Vegetable Sciences, China Agricultural University, Beijing 100193, China; Beijing Key Laboratory of Growth and Developmental Regulation for Protected Vegetable Crops, Department of Vegetable Sciences, China Agricultural University, Beijing 100193, China; Beijing Key Laboratory of Growth and Developmental Regulation for Protected Vegetable Crops, Department of Vegetable Sciences, China Agricultural University, Beijing 100193, China; Beijing Key Laboratory of Growth and Developmental Regulation for Protected Vegetable Crops, Department of Vegetable Sciences, China Agricultural University, Beijing 100193, China; Beijing Key Laboratory of Growth and Developmental Regulation for Protected Vegetable Crops, Department of Vegetable Sciences, China Agricultural University, Beijing 100193, China; Beijing Key Laboratory of Growth and Developmental Regulation for Protected Vegetable Crops, Department of Vegetable Sciences, China Agricultural University, Beijing 100193, China

## Abstract

Drought stress and lateral branches are both important factors affecting crop yield. Cucumber is a widely planted vegetable crop that requires a large amount of water during its production and varieties with few lateral branches are preferred. However, the mechanisms regulating cucumber drought tolerance and lateral branch development remain largely unclear. The MADS-box transcription factor *AGAMOUS-LIKE 16* (*CsAGL16*) was recently found to be a key positive regulator in cucumber shoot branching acting by stimulating abscisic acid (ABA) catabolism. In this study, we demonstrated that cucumber TCP interactor containing EAR motif protein 1 (CsTIE1) directly interacts with CsAGL16 at protein level and promotes lateral branch outgrowth through the CsAGL16–*CsCYP707A4* mediated ABA pathway in cucumber. Additionally, mutation of *CsAGL16* resulted in decreased drought tolerance, while overexpression of *CsAGL16* significantly enhanced drought tolerance in cucumber. Similarly, the drought resistance of *Cstie1* mutants was significantly reduced. However, overexpression of *CsAGL16* can enhance the drought tolerance of *Cstie1* mutants and promote their lateral branch outgrowth. These results indicated that the CsTIE1–CsAGL16 module was crucial for both lateral branch development and drought response, providing a strategy for cultivating drought-tolerant cucumber varieties with appropriate branch outgrowth.

## Introduction

Drought is one of the main environmental factors that affect the geographical distribution of plants, limit crop yields, and threaten food security. Due to global climate change, frequent extreme weather events such as droughts have accelerated land desertification, making droughts pose a more serious threat to agricultural production worldwide than ever before [1]. Therefore, analyzing the drought resistance mechanism of plants and improving their drought resistance is crucial for agricultural production.

The abscisic acid (ABA) signaling pathway plays an important role in plant response to drought stress. Drought stress can induce plant synthesis of ABA, thereby promoting the activation of plant drought response pathways and enhancing plant drought tolerance [2–5]. Related studies have shown that overexpression of ABA receptors such as *Pyrabattin Resistance 1* (*PYR1*) can improve crop drought resistance while increasing crop yield [6, 7]. The family of sucrose non-fermenting-1-related protein kinase 2 (SnRK2) is one of the core components of ABA signaling. Its family members SnRK2.2, SnRK2.3, and SnRK2.6 are activated by ABA and positively regulate ABA signaling and plant drought resistance [8–10].

When plants experience drought stress, in order to better survive and reproduce under drought conditions, in addition to activating drought response mechanisms, they also adjust their development processes, such as early flowering and slowing down lateral branch development [4, 11]. Lateral branches develop from axillary buds and have high plasticity. Lateral branches are an important agricultural trait and have been subjected to long-term selection and domestication. An appropriate number of lateral branches can help enhance the adaptability of plants to different environments and improve crop yield [12]. During axillary bud outgrowth, *BRANCHED1* (*BRC1*) is considered a central integration factor of multiple signaling pathways. Its expression is inhibited by the sugar and nitrogen signaling pathways, as well as hormone signaling pathways such as strigolactone, cytokinin, and gibberellin, but promoted by low red/far red light ratio [13]. In addition, BRC1 can inhibit axillary bud outgrowth by promoting the biosynthesis of ABA [14]. Studies have shown that TCP interactor containing EAR motif protein 1 (TIE1) can directly interact with BRC1 at the protein level to inhibit downstream gene expression and positively regulate lateral branch development [15, 16]. In addition, TIE1 can recruit TOPLESS (TPL) and TOPLESS-RELATED (TPR) proteins to inhibit the activity of TEOSINTE BRANCHED1/CYCLOIDEA/PCF (TCP) proteins and regulate *Arabidopsis* leaf development [17]. In cucumber, CsAGL16 has been shown to promote ABA catabolism in axillary buds by upregulating ABA 8′-hydroxylase gene *CsCYP707A4* expression, thereby positively regulating cucumber lateral branch outgrowth [18]. The above studies indicate that ABA plays an important role in regulating plant drought tolerance and lateral branch development, but its molecular mechanism for coordinating drought response and lateral branch development remains unclear.

Cucumber is a widely cultivated vegetable crop with high economic and nutritional value. The cultivation of cucumber requires a large amount of water, especially during the fruiting period. Water shortage can seriously affect cucumber yield. In addition, different market demands dictate varying requirements for cucumber shoot architecture. In Europe and the USA, processed cucumbers are often grown from varieties with multiple lateral branches, while in China fresh cucumber production typically favors varieties with few or no lateral branches [19, 20]. However, current research on the mechanism of drought resistance and lateral branch development regulation in cucumber remains relatively limited. Here we demonstrate that CsTIE1 directly interacts with CsAGL16 at the protein level and positively regulates cucumber lateral branch outgrowth through the CsAGL16–CsCYP707A4 pathway. In addition, *CsAGL16* and *CsTIE1* are positive regulatory factors for cucumber response to drought stress. In summary, this study demonstrates that *CsTIE1* and *CsAGL16* co-regulate cucumber lateral branch outgrowth and drought resistance, providing genetic resources for the cultivation of new varieties of drought-tolerant cucumber with few lateral branches.

**Figure 1:**
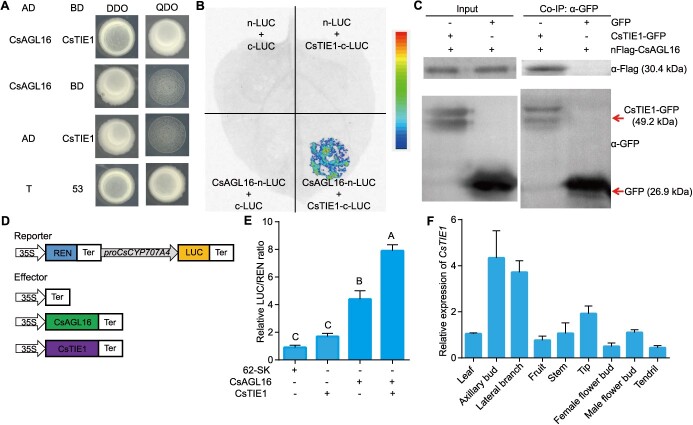
CsTIE1 interacts with CsAGL16 at the protein level. **A** Yeast two-hybrid assay. T-AD and 53-BD were used as positive controls. **B** Firefly LCI assay demonstrates the interaction between CsTIE1 and CsAGL16. When CsAGL16-nLUC and CsTIE1-cLUC were co-injected into *N. benthamiana* leaves, LUC activity could be detected, but not in other negative control groups. **C** The co-IP assay indicated that CsTIE1 interacts with CsAGL16. By immunoblotting, CsTIE1 and CsAGL16 proteins in total and precipitated proteins were detected using anti-GFP or anti-Flag antibody. **D** Schematic diagram of vector construction in dual-luciferase reporter analysis. The reporter was proCsCYP707A4:LUC. 35S::CsTIE1, 35S::CsAGL16, and 62-SK empty vectors were used as effectors. **E** Result of dual-luciferase reporter analysis. The empty vector 62-SK was used as the control. Different letters indicate significant differences (*P* < 0.01) through one-way ANOVA analysis with Tukey’s Studentized range (HSD) test. Values are mean ± standard deviation, *n* = 9. **F** Expression analyses of *CsTIE1* by qRT–PCR in different tissues. *CsUBI* was used as internal standard. Values are mean ± standard deviation, *n* = 3.

## Results

### CsTIE1 interacts with CsAGL16 at the protein level

Our previous study has shown that the MADS-box transcription factor *CsAGL16* positively regulates cucumber lateral branch outgrowth by promoting the expression of ABA 8′-hydroxylase gene *CsCYP707A4* [18]. In order to further explore the molecular mechanism of *CsAGL16* regulating cucumber lateral branch outgrowth, a yeast two-hybrid library screening was performed on CsAGL16. Through sequencing and BLAST analysis, a total of 55 candidate proteins that may interact with CsAGL16 were identified, among which the gene CsaV3_2G002110 was identified multiple times (Supplementary Data Table S1). Phylogenetic analysis showed that CsaV3_2G002110 has a close homologous relationship with *Arabidopsis* TCP interactor containing EAR motif protein 1 (TIE1) and TIE2, hence it is named CsTIE1 (Supplementary Data Fig. S1A). Subsequently, the direct interaction between CsTIE1 and CsAGL16 at the protein level was further validated through the yeast two-hybrid assay (Fig. 1A). The firefly luciferase complementation imaging (LCI) assay revealed that strong fluorescence signals could be detected when CsTIE1 co-transformed with CsAGL16 in *Nicotiana benthamiana* (Fig. 1B). Moreover, co-immunoprecipitation (co-IP) analysis showed that CsTIE1-GFP protein could be detected in the protein complex pulled down by anti-FLAG agarose (Fig. 1C), indicating that CsTIE1 and CsAGL16 indeed interact at the protein level.

**Figure 2:**
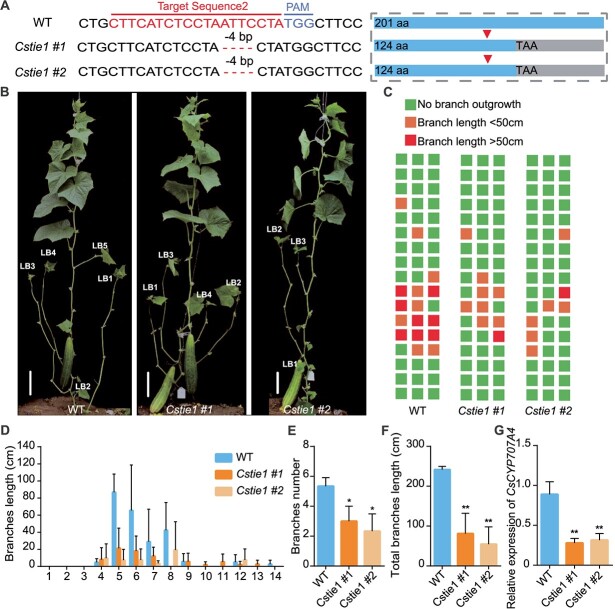
*Cstie1* mutants have shorter lateral branches. **A** The gene editing form of *Cstie1* mutants. The base sequence on the left displays the site and form of *CsTIE1* mutations. Red and blue sequences represent the target sequence and protospacer-adjacent motif (PAM), respectively. ‘-4 bp’ represents the number of deleted bases. The dashed box on the right shows that a mutation in *CsTIE1* caused translation to terminate prematurely. The triangle represents the location where the mutation occurred. aa, amino acids. **B** Representative images of WT and *Cstie1* mutants. LB, lateral branch. Scale bars, 10 cm. **C** Diagrammatic data on lateral branch length and position of WT and *Cstie1* mutants. Each plant is displayed as a column and each node is displayed as a square. **D** Average lateral branch length at each node in WT and *Cstie1* mutants. **E** Average branch number per plant of WT and *Cstie1* mutants. **F** Average total branch length per plant of WT and *Cstie1* mutants. **G** Relative expression of *CsCYP707A4* in WT and *Cstie1* mutants. Significance analysis was conducted with the two-tailed Student’s *t*-test (^*^P < 0.05 and ^**^P < 0.01). Values are mean ± standard deviation, *n* = 3.

To investigate the relationship between CsTIE1 and *CsCYP707A4*, a dual-luciferase reporter analysis was performed. The results showed that CsAGL16 can promote the expression of *CsCYP707A4*; unlike the direct transcription activation of Cs*CYP707A4* by CsAGL16, expression of *CsCYP707A4* was unaffected upon coexpression of CsTIE1. However, when CsTIE1 co-transformed with CsAGL16, the luciferase activity driven by the *CsCYP707A4* promoter significantly increased (Fig. 1D and E), indicating that CsTIE1 interaction with CsAGL16 promoted the transcriptional activation of *CsCYP707A4*-mediated ABA catabolism in cucumber lateral branch outgrowth. The expression pattern analysis results indicated that *CsTIE1* has a high expression level in axillary buds and lateral branches (Fig. 1F), suggesting that *CsTIE1* may play a role in regulating lateral branch development.

### *CsTIE1* mutation inhibits outgrowth of cucumber lateral branches

To explore the biological function of *CsTIE1*, homozygous loss-of-function mutants were obtained using CRISPR-Cas9-mediated gene editing technology in the cucumber inbred line R1461 (WT), and a total of nine independent stable genetic lines were obtained. Surprisingly, the identification results of the nine lines showed that they all lacked ATTC at the second target, leading to early termination of translation (Supplementary Data Fig. S1B). Since no other mutation forms were identified in the offspring, *Cstie1 #1* and *#2* were selected for subsequent phenotype analysis (Fig. 2A). Compared with WT, the number and length of lateral branches in the *Cstie1* mutants were significantly reduced (Fig. 2B and C). The statistical results indicate that, compared with WT, the average lateral branch length of each node in the *Cstie1* mutants is significantly shorter than that in WT (Fig. 2D). The *Cstie1* mutant has an average of 2.7 lateral branches per plant, while the WT has 5.3 branches per plant, which is 1.96 times that of the *Cstie1* mutants (Fig. 2E). The total lateral branch length per WT plant was 245 cm versus 85 and 65 cm in *Cstie1* mutants, i.e. the mutants had 65.3 and 73.4% decreased lateral branch length, respectively, compared with WT (Fig. 2F). Next, the expression level of *CsCYP707A4* was determined by quantitative real-time PCR (qRT–PCR). Compared with WT, the expression level of *CsCYP707A4* in the *Cstie1* mutants was reduced by ~66.6% (Fig. 2G), indicating that *CsTIE1* may regulate cucumber lateral branch outgrowth by promoting the transcriptional activation of *CsCYP707A4* via CsAGL16.

### Overexpression of *CsAGL16* can promote lateral branch outgrowth in *Cstie1* mutants

**Figure 3:**
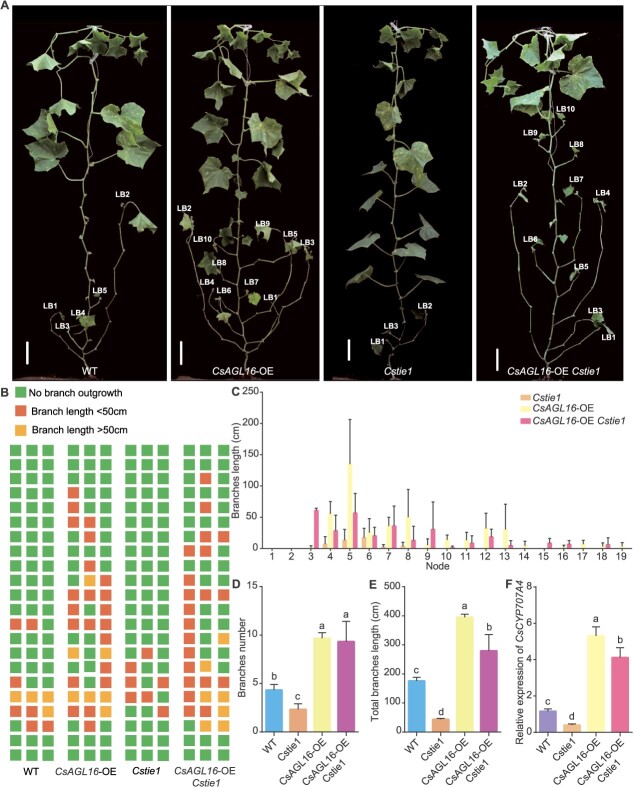
*CsAGL16*-OE *Cstie1* line phenotype analysis. **A** Representative images of WT, *Cstie1* mutants, *CsAGL16* overexpression (*CsAGL16*-OE) line, and a *CsAGL16*-OE *Cstie1* plant. LB, lateral branch. Scale bars, 10 cm. **B** Diagrammatic data on lateral branch length and position of WT, *Cstie1* mutants, *CsAGL16*-OE line, and *CsAGL16*-OE *Cstie1* plants. Each plant is displayed as a column and each node as a square. **C** Average lateral branch length at each node in *Cstie1* mutants and *CsAGL16*-OE line and *CsAGL16*-OE *Cstie1* plants. **D** Average branch number per plant of WT, *Cstie1* mutants, *CsAGL16*-OE line, and *CsAGL16*-OE *Cstie1* plants. **E** Average total branch length per plant of WT, *Cstie1* mutants, *CsAGL16*-OE line, and *CsAGL16*-OE *Cstie1* plants. **F** Relative expression of *CsCYP707A4* in WT, *Cstie1* mutants, *CsAGL16*-OE line, and *CsAGL16*-OE *Cstie1* plants. Different letters indicate significant differences (*P* < 0.05) through one-way ANOVA analysis with Tukey’s HSD test. Values are mean ± standard deviation, *n* = 3.

In order to analyze the interaction between *CsTIE1* and *CsAGL16* at the genetic level, we constructed *CsAGL16*-OE (*CsAGL16* overexpression in the cucumber inbred line R1461) *Cstie1* line by hybridization. Phenotypic observation revealed that the *CsAGL16*-OE *Cstie1* plants exhibited a significant increase in lateral branches, which was different from the *Cstie1* mutants with fewer lateral branches, and was similar to the *CsAGL16*-OE line (Fig. 3A and B). The average lateral branch length of each node in the *CsAGL16*-OE *Cstie1* plants was similar to that of the *CsAGL16*-OE line, but significantly longer than that of the *Cstie1* mutants (Fig. 3C). Each *Cstie1* mutant had ~2.3 lateral branches, which was only 53.8% of the WT. Each *CsAGL16*-OE *Cstie1* plant had an average of 9.3 lateral branches, which was 2.2- and 4.0-fold that of WT and *Cstie1* mutants, respectively, similar to the *CsAGL16*-OE line (Fig. 3D). This resulted in a significant increase in the average total lateral branch length (279.8 cm) of the *CsAGL16*-OE *Cstie1* plants, which was 1.6 times and 6.4 times that of the WT and *Cstie1* mutants, respectively, reaching 70.8% of the *CsAGL16*-OE line (Fig. 3E). In addition, overexpression of *CsAGL16* significantly increased the expression level of *CsCYP707A4* in *Cstie1* mutants (Fig. 3F). The above results indicated that overexpression of *CsAGL16* significantly promotes lateral branch outgrowth in *Cstie1* mutants, suggesting that *CsTIE1* regulates cucumber lateral branch outgrowth through *CsAGL16*.

### *CsAGL16* is a positive regulatory factor for drought tolerance in cucumber

**Figure 4:**
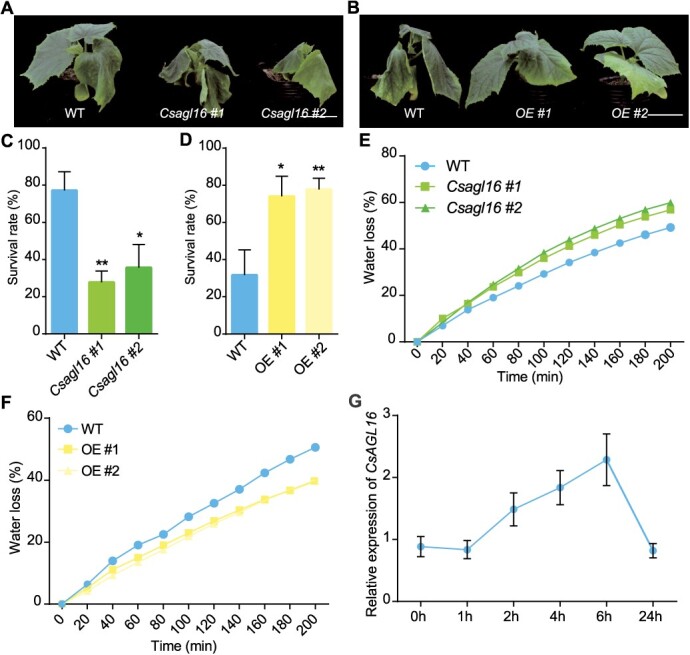
Drought tolerance analyses of *CsAGL16* transgenic lines. **A** and **B** Representative images of WT, *Csagl16* mutants (A) and OE lines (B) under drought treatment. Scale bars, 2 cm. **C** and **D** Survival rate of WT, *Csagl16* mutants (**C**) and OE lines (**D**) after drought treatment. **E** and **F** Analysis of water loss rate of WT, *Csagl16* mutants (**E**), and OE lines (**F**). OE, *CsAGL16* overexpression line. **G** Expression analyses of *CsAGL16* by qRT–PCR under drought treatment in cucumber. Significance analysis was conducted with the two-tailed Student’s *t*-test (^*^P < 0.05 and ^**^P < 0.01). Values are mean ± standard deviation, *n* = 3.

Studies in *Arabidopsis* have shown that *AGL16* negatively regulates drought tolerance by regulating stomatal density and ABA accumulation [21], and the absence of *AGL16* also increases *Arabidopsis* tolerance to salt stress [22]. To investigate whether *CsAGL16* plays a role in cucumber response to drought stress, 25-day-old cucumber seedlings were subjected to drought treatment. After drought treatment, the *Csagl16* mutants showed more severe wilting compared with WT, while the wilting degree of the OE (*CsAGL16* overexpression) lines was lighter (Fig. 4A and B). After continuing drought treatment for 2–3 days followed by a full irrigation recovery period of 3 days, most plants of the *Csagl16* mutants did not recover (survival rates were only 27.8–35.7%), while the corresponding WT showed a survival rate of 77.2% (Fig. 4C). The survival rate of *CsAGL16* overexpression lines (74.1–77.8%) significantly increased after rehydration, being 2.4 times that of the WT (Fig. 4D). These results indicated that overexpression of *CsAGL16* could improve the drought resistance of cucumber seedlings, while its mutation has the opposite effect. This led us to hypothesize that the water loss rate of *CsAGL16* transgenic plants may differ from that of WT. Subsequently, the water loss rates of different lines were tested within 200 min. As time passed the water loss rate of different plants gradually increased, but the water loss rate of *Csagl16* mutants was always higher than that of WT (Fig. 4E), while that of the *CsAGL16*-OE lines was significantly lower than that of WT (Fig. 4F), indicating that *CsAGL16* regulates cucumber drought resistance by affecting water loss.

### *CsAGL16* regulates drought tolerance in cucumber by influencing stomatal closure and root development

**Figure 5:**
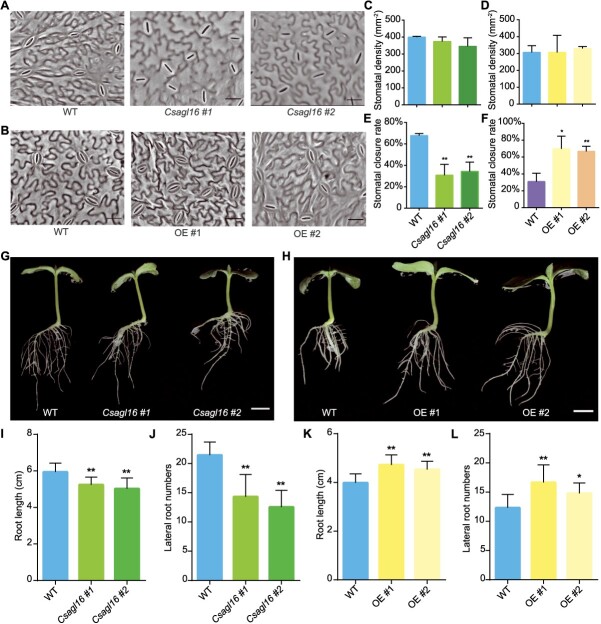
Phenotypic analysis of stomata and roots in *CsAGL16* transgenic lines. **A** and **B** Representative images of stomata on the abaxial epidermis of WT, *Csagl16* mutants (**A**), and OE lines (**B**). Scale bars, 20 μm. **C** and **D** Statistical analysis of stomatal density of WT, *Csagl16* mutants (**C**), and OE lines (**D**). **E** and **F** Statistical analysis of the rate of closed stomata in WT, *Csagl16* mutants (**E**), and OE lines (**F**). **G** and **H** Representative images of roots of WT, *Csagl16* mutants (**G**), and OE lines (**H**) from 5-day-old seedlings. Scale bars, 1 cm. **I** and **J** Statistical analysis of root length (**I**) and lateral root number (**J**) of *Csagl16* mutants from 5-day-old seedlings. **K** and **L** Statistical analysis of root length (**K**) and lateral root number (**L**) of OE lines from 5-day-old seedlings. OE, *CsAGL16* overexpression line. Significance analysis was conducted with the two-tailed Student’s *t*-test (^*^P < 0.05 and ^**^P < 0.01). Values are mean ± standard deviation, *n* = 3 in **C**–**F** and *n* = 9 in **I**–**L**.

Due to the transpiration of stomata on the leaf being the main source of plant water loss, the stomatal density on the leaf abaxial side of different lines was tested. Under sufficient water conditions, there was no significant difference in stomatal density between *CsAGL16* mutants and overexpression lines compared with WT (Fig. 5A–D). Next, the number of closed stomata in different lines was determined after drought treatment. The observation results indicate that, compared with WT, the *Csagl16* mutants had fewer closed stomata but OE lines had more closed stomata (Fig. 5A and B). The statistical results showed that the stomatal closure rate of *Csagl16* mutants was only 48.0% of that of WT, while that of the OE lines was 2.2-fold that of WT (Fig. 5E and F), indicating that *CsAGL16* promoted stomatal closure in cucumber under drought conditions.

In addition to the water loss rate, water absorption is also an important factor affecting plant drought resistance. The root system structure plays a crucial role in drought stress, and a well-developed root system helps to increase water absorption, thereby improving plant tolerance to drought [11, 23]. Next, the root system of 5-day-old seedlings of *CsAGL16* transgenic lines was analyzed. Compared with WT, the root length and lateral root number of the *Csagl16* mutants were significantly reduced, by 6.2 and 37.3% respectively (Fig. 5G, I, and J), while the OE lines showed significant increases in root length and lateral root number of 8.6 and 27.5% respectively (Fig. 5H, K, and L). As plants grow, the differences in root length and lateral root number between *CsAGL16* transgenic lines and WT become more significant (Supplementary Data Fig. S2). The above results indicated that *CsAGL16* improves the drought resistance of cucumber seedlings by promoting stomatal closure and root growth.

### *CsAGL16* promotes the clearance of oxidants in cucumber plants under drought stress

Drought can promote the production of reactive oxygen species (ROS) in organelles, while ROS can act as signaling molecules to induce programmed cell death. Therefore, the increase of ROS is not conducive to plant growth and production [24]. To counteract the damage caused by ROS, plants express various antioxidant enzymes, such as superoxide dismutase (SOD) and peroxidase (POD) [24, 25]. Studies in cucumber have shown that when cucumber seedlings are subjected to drought stress, the levels of these enzymes significantly increase [26, 27].

**Figure 6:**
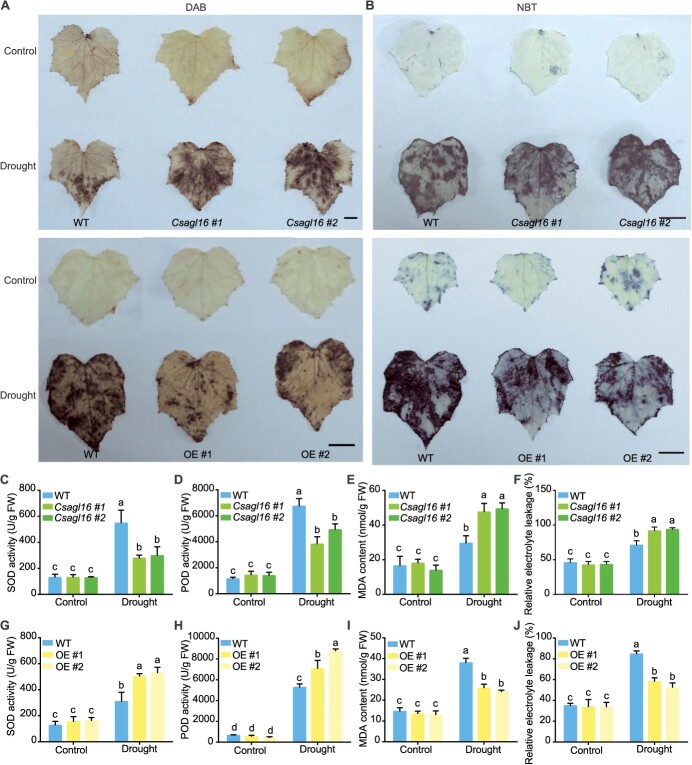
*CsAGL16* positively regulates cucumber response to drought. **A** H_2_O_2_ content was determined by DAB staining. **B** SOD enzyme activity was determined using NBT staining. **C** SOD enzyme activity of *Csagl16* mutants. **D** POD activity of *Csagl16* mutants. **E** MDA content of *Csagl16* mutants. **F** Relative electrolyte leakage of *Csagl16* mutants. **G** SOD enzyme activity of OE lines. **H** POD activity of OE lines. **I** MDA content of OE lines. **J** Relative electrolyte leakage of OE lines. OE, *CsAGL16* overexpression line. Different letters indicate significant differences (*P* < 0.05) through one-way ANOVA analysis with Tukey’s HSD test. Values are mean ± standard deviation, *n* = 3.

Firstly, the H_2_O_2_ content and SOD enzyme activity in the leaf of *CsAGL16* transgenic plants under normal conditions and after drought treatment were determined using diaminobenzidine tetrahydrochloride (DAB) and nitroblue tetrazolium (NBT) staining methods, respectively. Under normal conditions, whether using DAB staining or NBT staining, there was no difference between *CsAGL16* transgenic lines and WT (Fig. 6A and B). After drought treatment, compared with WT, the leaves of the *Csagl16* mutants showed darker colors while the OE lines had lighter colors (Fig. 6A and B), indicating that the *Csagl16* mutants had higher H_2_O_2_ content and lower SOD enzyme activity, while the OE lines had the opposite phenotype. Subsequently, the enzyme activities of SOD and POD were determined quantitatively and the results showed that the enzyme activities of *Csagl16* mutants were significantly reduced after drought treatment (Fig. 6C and D). In addition, compared with WT, the malondialdehyde (MDA) content and relative electrolyte leakage of the *Csagl16* mutants significantly increased after drought treatment (Fig. 6E and F), indicating that the membrane damage of the *Csagl16* mutants was more severe after drought stress. The analysis of SOD and POD enzyme activity, MDA content, and relative electrolyte leakage of OE lines after drought treatment showed opposite results to the *Csagl16* mutants (Fig. 6G–J), indicating that increasing the expression level of *CsAGL16* helps cucumber seedlings to quickly clear ROS under drought conditions.

### Mutation of *CsTIE1* leads to reduced drought resistance in cucumber seedlings

**Figure 7:**
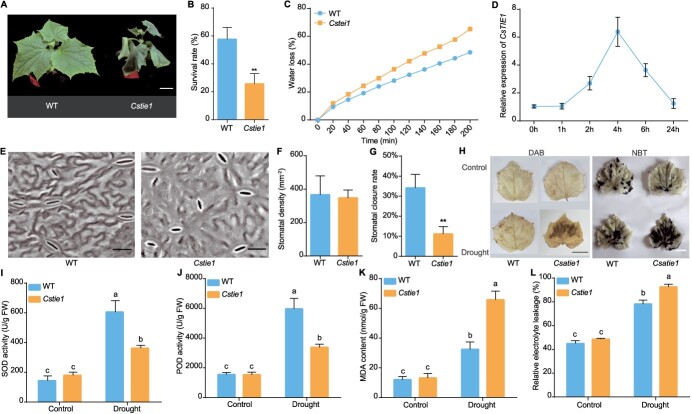
Drought tolerance analyses of *Cstie1* mutants. **A** Representative images of WT and *Cstie1* mutants under draught treatment. Scale bar, 2 cm. **B** Survival rate of WT and *Cstie1* mutants after drought treatment. **C** Analysis of water loss rate of WT and *Cstie1* mutants. **D** Expression analyses of *CsTIE1* by qRT–PCR under drought treatment in cucumber. **E** Representative images of stomata on the abaxial epidermis of WT and *Cstie1* mutants. Scale bars, 20 μm. **F** Statistical analysis of stomatal density of WT and *Cstie1* mutants. **G** Statistical analysis of the rate of closed stomata in WT and *Cstie1* mutants. Significance analysis was conducted with the two-tailed Student’s *t*-test (^**^P < 0.01) in **B**, **F** and **G**. **H** H_2_O_2_ content and SOD enzyme activity were determined by DAB and NBT staining, respectively. **I** SOD enzyme activity of *Cstie1* mutants. **J** POD activity of *Cstie1* mutants. **K** MDA content of *Cstie1* mutants. **L** Relative electrolyte leakage of *Cstie1* mutants. Different letters indicate significant differences (*P* < 0.05) through one-way ANOVA analysis with Tukey’s HSD test in **I**–**L**. Values are mean ± standard deviation, *n* = 3.

Afterwards, we investigated whether *CsTIE1* also participates in the drought response of cucumber seedlings. After 8 days of drought treatment, the *Cstie1* mutants showed varying degrees of wilting, while the WT remained in a normal growth state (Fig. 7A). Continuing drought for 4–5 days, followed by a full irrigation recovery period of 3 days, 66.7% of WT seedlings returned to normal growth, while only 33.3% of *Cstie1* mutants did so (Fig. 7B). Within 200 min, the water loss rate of *Cstie1* mutants was significantly higher than that of WT (Fig. 7C), and their root length and lateral root number were significantly lower than WT (Supplementary Data Fig. S3), indicating that the poor drought resistance of *Cstie1* mutants was due to its high water loss rate and low water absorption. In addition, qRT–PCR analysis showed that drought treatment significantly induced the expression of *CsTIE1* (Fig. 7D). Analysis of stomatal phenotype revealed that the stomatal density of *Cstie1* mutants was similar to that of WT. However, under drought stress *Cstie1* mutants had fewer stomata closed, with a stomatal closure rate only 33.8% of that in WT (Fig. 7E–G). The DAB and NBT staining results showed that, compared with WT, after drought treatment *Cstie1* mutants had higher H_2_O_2_ content and lower SOD enzyme activity (Fig. 7H). The quantitative analysis results also showed that after drought treatment the enzyme activities of SOD and POD in *Cstie1* mutants were significantly lower than those in WT (Fig. 7I and J), leading to an increase in MDA levels (Fig. 7K) and severe damage to the membrane system, resulting in ion efflux within the cell and an increase in relative electrolyte leakage (Fig. 7L).

### Overexpression of *CsAGL16* can enhance the drought tolerance of *Cstie1* mutants

**Figure 8:**
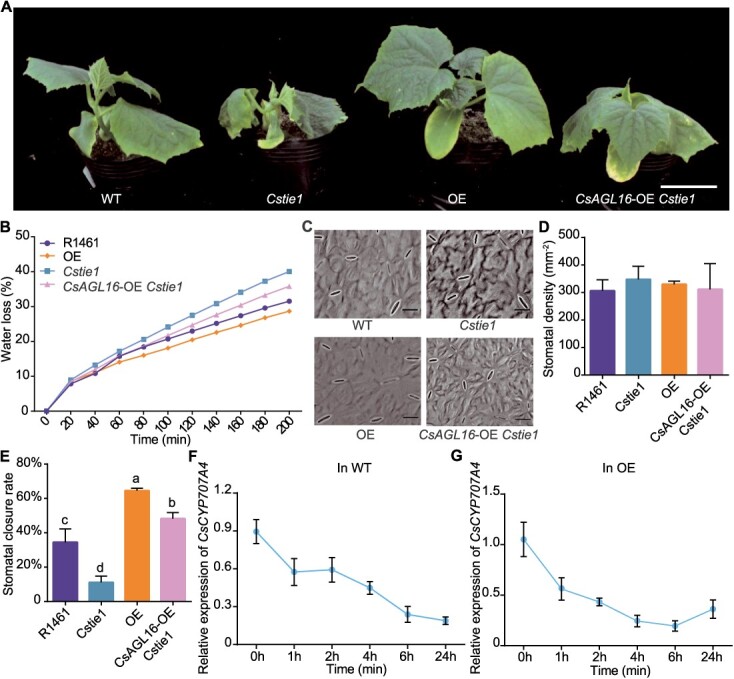
Drought tolerance analyses of *CsAGL16*-OE *Cstie1* line. **A** Representative images of WT, *Cstie1* mutants under draught treatment, OE plants, and *CsAGL16*-OE *Cstie1* line. Scale bar, 2 cm. **B** Analysis of water loss rate of WT, *Cstie1* mutants, OE plants, and *CsAGL16*-OE *Cstie1* line. **C** Representative images of stomata on the abaxial epidermis of WT, *Cstie1* mutants, OE plants, and *CsAGL16*-OE *Cstie1* line. Scale bars, 20 μm. **D** Statistical analysis of stomatal density of WT, *Cstie1* mutants, OE plants, and *CsAGL16*-OE *Cstie1* line. **E** Statistical analysis of the rate of closed stomata in WT, *Cstie1* mutants, OE plants, and *CsAGL16*-OE *Cstie1* line. **F** Analysis of changes in *CsCYP707A4* expression levels in WT under different drought treatment times. **G** Analysis of changes in *CsCYP707A4* expression levels in OE line under different drought treatment times. OE, *CsAGL16* overexpression line. Different letters indicate significant differences (*P* < 0.05) through one-way ANOVA analysis with Tukey’s HSD test in **D and E**. Values are mean ± standard deviation, *n* = 3.

Next, in order to analyze the interaction between CsAGL16 and CsTIE1 in regulating cucumber drought tolerance, the drought tolerance of the *CsAGL16*-OE *Cstie1* line was analyzed. After drought treatment, the wilting degree of the *CsAGL16*-OE *Cstie1* line was weaker than that of *Cstie1* mutants, but more severe than that of *CsAGL16*-OE plants (Fig. 8A). The analysis of water loss rate showed that the water loss rate of the *CsAGL16*-OE *Cstie1* line was higher than that of WT, but lower than that of *Cstie1* mutant (Fig. 8B). Analysis of stomatal phenotype revealed that there was no significant difference in stomatal density between the *CsAGL16*-OE *Cstie1* line and WT, but there was a change in stomatal closure rate (Fig. 8C-E). When subjected to drought stress, 48.2% of the stomata in the *CsAGL16*-OE *Cstie1* line were closed, a significantly lower percentage than the 64.5% in the OE plants, but significantly higher than the 11.2% in the *Cstie1* mutants (Fig. 8E). These results indicate that increasing the expression level of *CsAGL16* can enhance the drought tolerance of *Cstie1* mutants.

In addition, *CsAGL16* OE lines had lower ABA levels in axillary buds, but their drought tolerance was significantly enhanced. In order to investigate the reasons for these results, we conducted further experiments to analyze the molecular mechanism involved. The expression levels of *CsCYP707A4* in WT and OE plants were determined by qRT–PCR after drought treatment for different periods of time. The results showed that the expression level of *CsCYP707A4* continued to decrease with increasing drought treatment time in both WT and OE lines (Fig. 8F and G), indicating that drought stress not only inhibited the expression of *CsCYP707A4*, but also inhibited the transcriptional promotion of *CsCYP707A4* by CsAGL16.

## Discussion

Lateral branches are an important component of shoot architecture, affecting the planting density and yield of crops. Studies in *Arabidopsis* and cotton have shown that TIE1 inhibits downstream gene expression by directly interacting with BRC1 at the protein level, thereby regulating lateral branch development. Mutations in *TIE1* result in reduced shoot activity and branching [15, 16]. In addition, it has been demonstrated that *TIE1* regulates *Arabidopsis* root development by inhibiting the key transcription factor type-B *Arabidopsis response regulator 1* (*ARR1*) in the cytokinin signaling pathway. The root length and lateral root number of the *tie1-1 tie2-1* double mutant were significantly reduced [28]. Here, our results indicated that CsTIE1 directly interacts with the MADS-box transcription factor CsAGL16 at the protein level (Fig. 1) and positively regulates cucumber lateral branch outgrowth (Fig. 2B–F). Unlike *TIE1* in *Arabidopsis*, which inhibits the expression of the TCP family by recruiting TPL and TPR proteins [17], our results showed that the interaction between CsTIE1 and CsAGL16 promotes transcriptional activation of *CsCYP707A4* via CsAGL16 (Fig. 1D), demonstrating that the molecular mechanism of cucumber *CsTIE1* regulating lateral branch development is different from that of its homologous gene in *Arabidopsis*. We speculate that there may be two possible reasons why TIE1 exhibits transcriptional inhibitory activity in *Arabidopsis* and transcriptional promoting activity in cucumber: on the one hand, TIE1 may have undergone functional differentiation from *Arabidopsis* to cucumber; on the other hand, it may be due to differences in the interacting proteins.

Previous studies have shown that CsAGL16 promotes the expression of the ABA 8′-hydroxylase gene *CsCYP707A4*, leading to a decrease in ABA levels in axillary buds and promoting cucumber axillary bud outgrowth [18]. The qRT–PCR detection results showed that the expression level of *CsCYP707A4* was significantly reduced in the axillary buds of *Cstie1* mutants (Fig. 2G). In addition, the lateral branch length and number of *CsAGL16*-OE *Cstie1* plants significantly increased, which is opposite to the phenotype of *Cstie1* mutant, but similar to *CsAGL16*-OE lines (Fig. 3), indicating that *CsTIE1* functions through the CsAGL16–*CsCYP707A4* pathway during lateral branch outgrowth in cucumber.

Drought is a major factor limiting agricultural productivity. Cucumber and other vegetable crops are typically varieties with poor drought resistance and require a large amount of water during the production process [11]. Therefore, improving the drought resistance of excellent germplasm through molecular breeding technology is of great significance for increasing the yield of crops, especially vegetable crops. Studies have shown that *AGL16* not only participates in flowering regulation, but also negatively regulates drought tolerance in *Arabidopsis* [21, 29]. In rice, the *AGL16*-homologous gene *OsMADS57* coordinates both tillering development and chilling tolerance [30]. Here, we demonstrated that *CsAGL16* is a positive regulatory factor for drought tolerance in cucumber (Fig. 4), which is contrary to the negative regulation of drought tolerance in *Arabidopsis* by *AGL16* [21]. Unlike the results in *Arabidopsis* showing that *AGL16* mutation leads to decreased stomatal density and stomatal opening under drought conditions [21], *CsAGL16* mutation does not affect stomatal density, but displays increased stomatal opening and inhibited root development (Fig. 5), which results in the role of *CsAGL16* in drought resistance being opposite in cucumber compared with *Arabidopsis*. Further research is needed to explore the underlying molecular mechanisms of this difference. This is also different from the function of *OsMADS57* in rice, indicating that *AGL16* and its homologous genes may have undergone functional differentiation in different species.

There are four *TIE*-paralogous genes in *Arabidopsis*, among which *TIE1* has been shown to be involved in regulating the development of *Arabidopsis* leaves and lateral branches [16, 17]. In addition, it has been demonstrated that *TIE1* and *TIE2* positively regulate the development of *Arabidopsis* root and exhibit functional redundancy [28]. However, it remains unclear whether *TIE* and its homologous genes are involved in the plant drought stress response. Our evidence suggests that the homologous gene *CsTIE1* positively regulates the drought resistance of cucumber seedlings by affecting stomatal density and root development (Fig. 7 and Supplementary Data Fig. S3). In addition, qRT–PCR analysis showed that both *CsTIE1* and *CsAGL16* were significantly induced by drought (Figs 4G and  7D), while *CsCYP707A4* was continuously inhibited by drought (Fig. 8F and G), indicating that the expression activation of *CsCYP707A4* by CsTIE1 and CsAGL16 was strongly inhibited under drought stress, and leading to the transformation of cucumber from growth and development to defense drought stress. However, the molecular mechanism underlying this transformation is still unclear and further research is needed.

**Figure 9:**
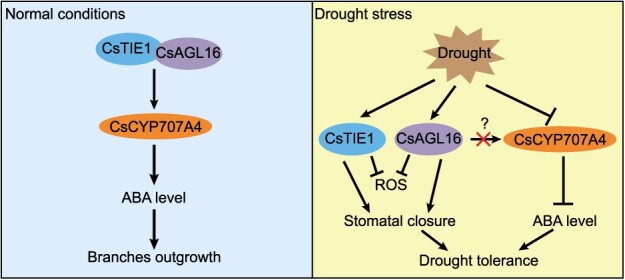
CsTIE1 interacts with CsAGL16 to coordinate cucumber branch outgrowth and drought tolerance. Under normal conditions, CsTIE1 interacts with CsAGL16 to promote transcriptional activation of *CsCYP707A4* by CsAGL16, leading to a decrease in ABA levels in axillary buds and promoting lateral branch outgrowth. When cucumber seedlings were subjected to drought stress, drought stress could induce the expression of CsTIE1 and CsAGL16, thereby inhibiting ROS levels and promoting stomatal closure to reduce water loss. On the other hand, drought inhibits the expression of *CsCYP707A4* and suppresses the transcriptional activation of *CsCYP707A4* by CsAGL16 through an unknown mechanism, resulting in a decrease in *CsCYP707A4* expression levels and an increase in ABA levels, thereby improving the drought tolerance of cucumber seedlings.

In summary, this study demonstrated that CsTIE1 interacts directly with CsAGL16 at the protein level and promotes the transcriptional activation of *CsCYP707A4* by CsAGL16, thereby positively regulating cucumber lateral branch outgrowth. Moreover, both *CsTIE1* and *CsAGL16* positively regulate the drought resistance of cucumber seedlings by promoting stomatal closure and root development and clearing ROS (Fig. 9). This study analyzed the mechanisms by which *CsTIE1* and *CsAGL16* regulate cucumber lateral branch outgrowth and drought resistance, providing a theoretical basis and genetic resources for cultivating new drought-resistant cucumber varieties with few lateral branches suitable for cultivation in China.

## Materials and methods

### Plant materials and planting

Cucumber (*Cucumis sativus* L.) inbred line R1461 was used for gene expression analysis and construction of *CsTIE1* gene-edited lines. The *CsAGL16*-OE lines and *Csagl16* mutants were previously constructed in the laboratory [18]. Cucumber seeds were germinated in the dark at 28°C and then planted in seedling bowls. Plants were cultivated in an illuminating incubator (25°C, 16 h of light/8 h of darkness) for ~3 weeks, then transplanted to a standard greenhouse located at China Agricultural University; water and fertilizer management and pest control were carried out according to standard conditions.

*Nicotiana benthamiana* seeds were sown directly into seedling bowls, and after the cotyledons were fully opened, seedlings with good and consistent growth were selected for separate cultivation. When grown to four or five true leaves at 25°C under 16 h of light/8 h of darkness, plants with good growth were selected for subsequent biochemical analysis.

### Genetic transformation of cucumber

The CRISPR-P2.0 website (http://crispr.hzau.edu.cn/CRISPR2/) was used to design the specific sgRNA target sites targeting the *CsTIE1* coding region. Then, we selected two specific sgRNA target sites and cloned a DNA fragment containing the two sites using the four-primer amplification method, and constructed the above DNA fragment into the pKSE402 vector using the BsaI site to obtain the final target vector [31, 32]. Then, the cucumber cotyledon genetic transformation method mediated by *Agrobacterium tumefaciens* was used for cucumber genetic transformation. Positive transgenic seedlings were screened using GFP fluorescent screening markers carried by the pKSE402 vector, and the editing mode of the *CsTIE1* gene in positive seedlings was detected using DNA sequencing technology. The edited seedlings were planted in a greenhouse and homozygous gene-edited plants of *CsTIE1* were obtained through self-pollination. The primer information is listed in Supplementary Data Table S2.

### RNA extraction and expression analysis

Fresh axillary buds or young leaves taken from the top of plants were placed in a 1.5-mL RNA-free centrifuge tube, and quickly placed in liquid nitrogen. Next, the samples were rapidly ground into powder under freezing conditions and total RNA was extracted using the Eastep^®^ Super Total RNA Extraction Kit (Promega, Madison, WI, USA). Then, the FastKing gDNA Dispelling RT SuperMix Kit (Tiangen, Beijing, China) was used to reverse-transcribe the total RNA into cDNA. The above cDNA was used as a template and qRT–PCR analysis was performed using TB Green^®^ Premix Ex Taq™ II (Takara, Kyoto, Japan) on a CFX384 Real-Time PCR System (Bio-Rad, Hercules, CA, USA). The cucumber *Ubiquitin* gene was used as an internal reference gene [33]. The primer information is listed in Supplementary Data Table S2.

### Yeast two-hybrid assay

The full-length coding sequence of *CsAGL16* was cloned and constructed into vector pGADT7 through EcoRI and BamHI sites. At the same time, the full-length coding sequence of *CsTIE1* was cloned and constructed into the vector pGBKT7 through XmaI and BamHI sites. The recombinant plasmid was transformed into yeast strain Y2HGold according to the Yeast Protocols Handbook (Clontech). The primer information is listed in Supplementary Data Table S2.

### Dual-luciferase reporter analysis

The full-length coding sequences of *CsTIE1* and *CsAGL16* were cloned by PCR and constructed into the pGreenII 62-SK vector as effectors. The 2000-bp promoter sequence of *CsCYP707A4* was constructed into the pGreenII 0800-LUC vector as a reporter. Then, the above-mentioned recombinant plasmids were transformed into *A. tumefaciens* GV3101 (pSoup-p19). Next, *A. tumefaciens* cells containing effectors and reporter were mixed in a 9:1 ratio and injected into *N. benthamiana* leaves, followed by cultivation under dark conditions for 24 h under normal conditions (16 h of light/8 h of darkness) for 48 h. Then the activities of firefly luciferase (LUC) and *Renilla reiformis* luciferase (REN) were detected using the Promega Dual-Luciferase Reporter Assay System kit. The primer information is listed in Supplementary Data Table S2.

### Co-immunoprecipitation analysis

For co-IP the *CsTIE1* coding sequence without the termination codon was constructed into pCAMBIA1300-GFP vector to obtain the CsTIE1 protein expression vector CsTIE1-GFP. The previously constructed *CsAGL16* overexpression vector (*Pro35S:nflag-CsAGL16*) was used as the CsAGL16 protein expression vector [18]. The protein expression vectors were transformed into *A. tumefaciens* GV3101 strain and co-injected with p19 *A. tumefaciens* into *N. benthamiana* leaves. After 48 h, samples were collected and protein was enriched using anti-GFP nanobody-coated agarose beads (KT-Health, China, catalog number KTSM1301). Next, the protein was denatured at 100°C and separated by SDS–PAGE, and the target protein was detected using immunoblotting with anti-GFP (TransGen Biotech, Beijing, China, catalog number HT801) or anti-FLAG (Sigma–Aldrich, Burlington, MA, USA; catalog number F3165) antibodies. The primer information is listed in Supplementary Data Table S2.

### Drought treatment

In the analysis of cucumber drought tolerance, watering was stopped when cucumber seedlings had grown to the 25th day, and the phenotype was observed and statistically analyzed when the seedlings wilted. Thirty-two plants per line were treated, with three repetitions.

In the experiment to detect whether the target gene is induced by drought, 3 days after watering had stopped, 25-day-old cucumber seedlings were watered with a sufficient amount of 10% (w/v) mannitol solution [34], and samples (second true leaf) were taken after watering for 0, 1, 2, 4, 6, and 24 h. After freezing the sample in liquid nitrogen, it was stored at −80°C for RNA extraction and target gene expression analysis.

### Measurement of H_2_O_2_ levels and antioxidant enzyme activity

According to the manufacturer’s protocols, the H_2_O_2_ levels, MDA content, and the activities of SOD and POD were measured in different plants under normal conditions and after drought treatment using detection kits (Solarbio, China). The contents of H_2_O_2_ and ROS were determined using DAB and NBT staining, respectively.

## Supplementary Material

Web_Material_uhae279

## Data Availability

The data that support the findings of this study have been included in the supplementary data.
