# Supplementary material for: The CsTIE1–CsAGL16 module regulates lateral branch outgrowth and drought tolerance in cucumber
Source: Hortic Res. 2024 Oct 2;12(1):uhae279. doi: 10.1093/hr/uhae279 (PMC11756290; doi:10.1093/hr/uhae279)
Supplement: Web_Material_uhae279 [file web_material_uhae279.zip › Supplemental data.docx]

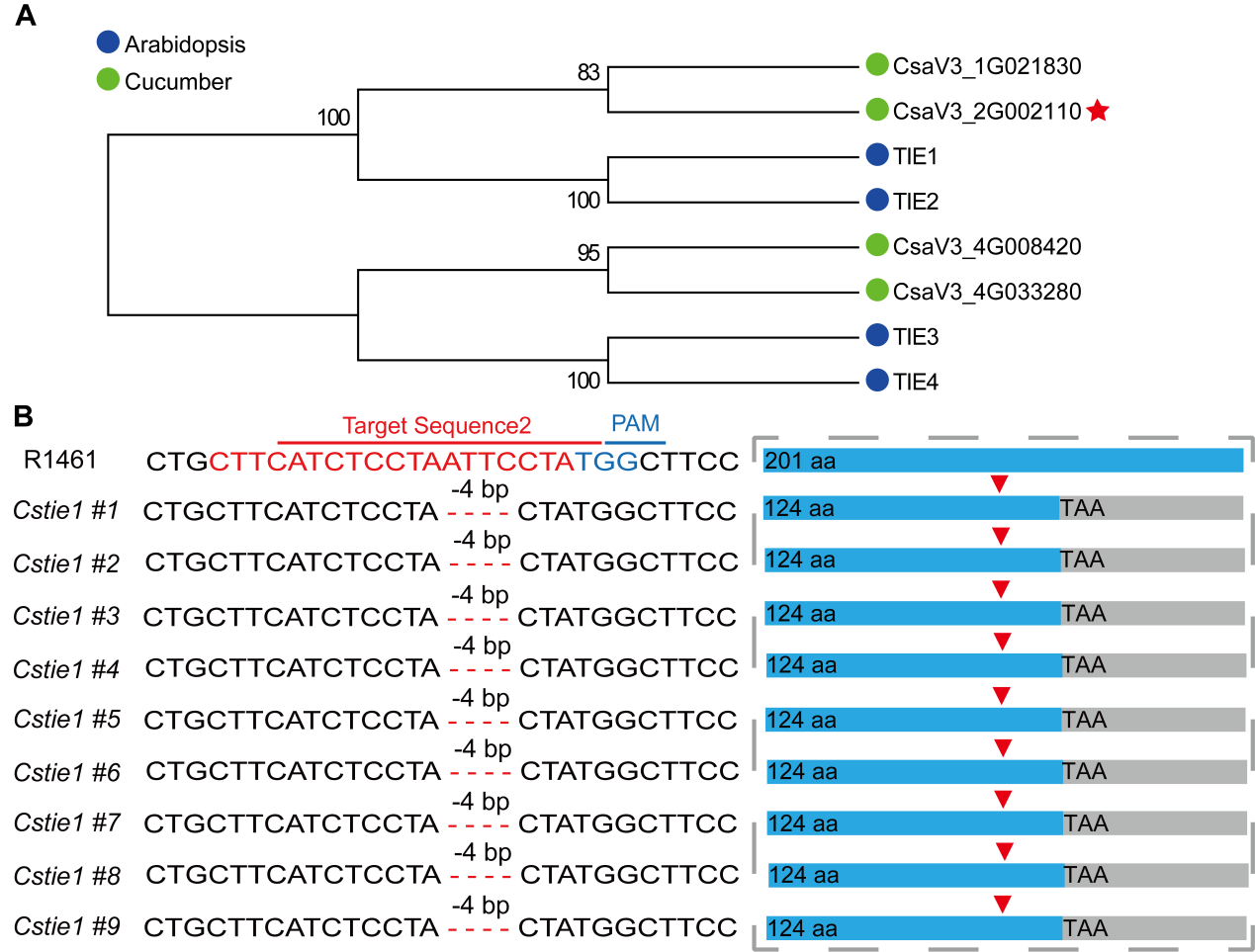


**Supplemental Figure S1 Phylogenetic tree analysis of *CsTIE1* and gene editing form of *Cstie1* mutants. A** Phylogenetic tree analysis of *CsTIE1* homolog genes. **B** The gene editing form of *Cstie1* mutants. The base sequence on the left displays the site and form of *CsTIE1* mutation. The red and blue sequences represent target sequence and protospacer-adjacent motif (PAM), respectively. “-4bp” represents the number of deleted bases. The dashed box on the right shows that a mutation in *CsTIE1* caused the translation to terminate prematurely. The triangle represents the location where the mutation occurred. aa, amino acid.


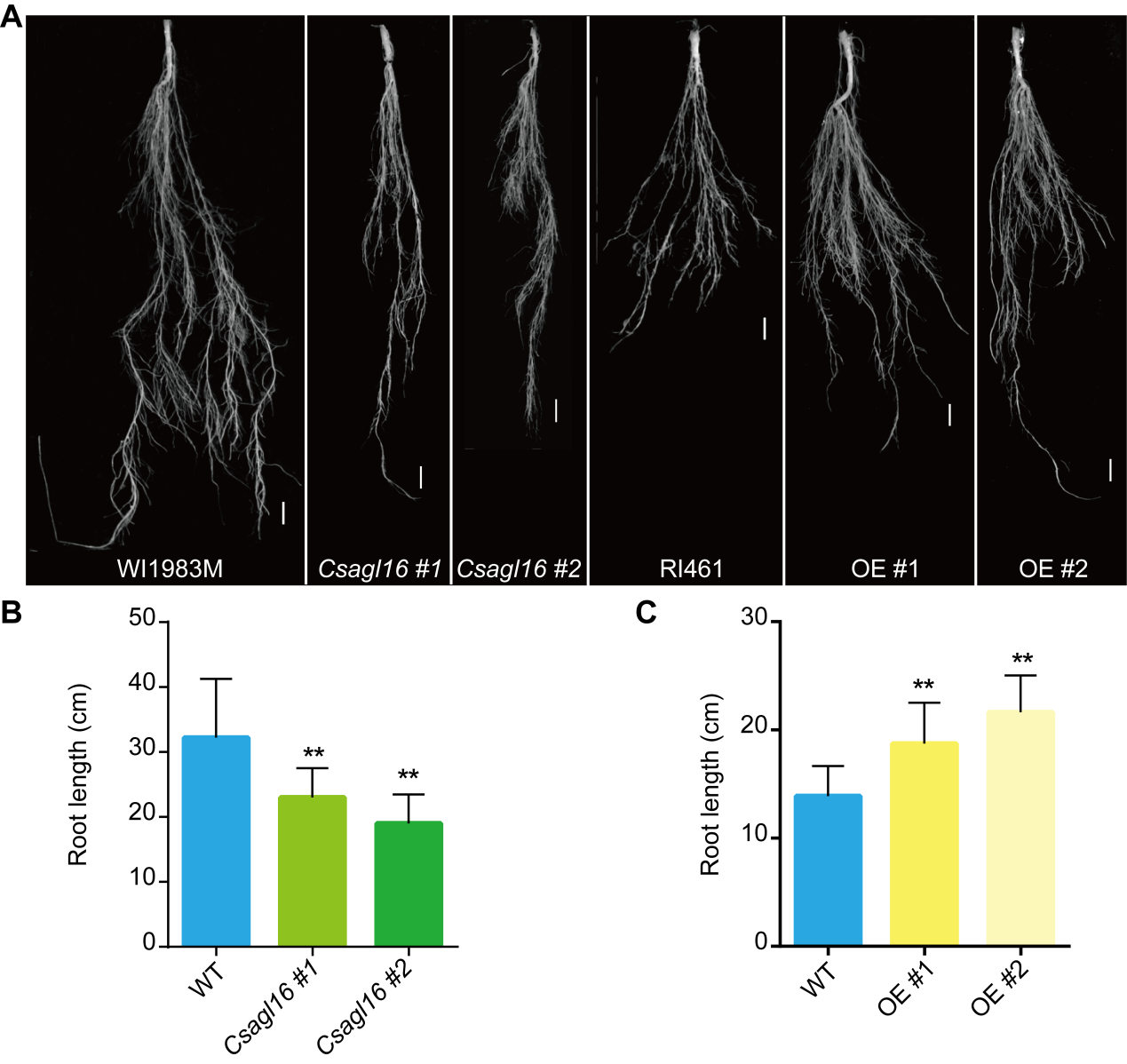


**Supplemental Figure S2 Root length analysis of *CsAGL16* transgenic lines. A** Representative images of roots of WT (WI1983M and R1461), *Csagl16* mutants and OE lines 20-day-old seedlings. Scale bars, 1 cm. **B** and **C** Statistical analysis of root length of *Csagl16* mutants (B) and OE plants (C). Significance analysis was conducted with the two-tailed Student’s *t*-test (** *p* < 0.01). Values are means ± SD, n = 10.


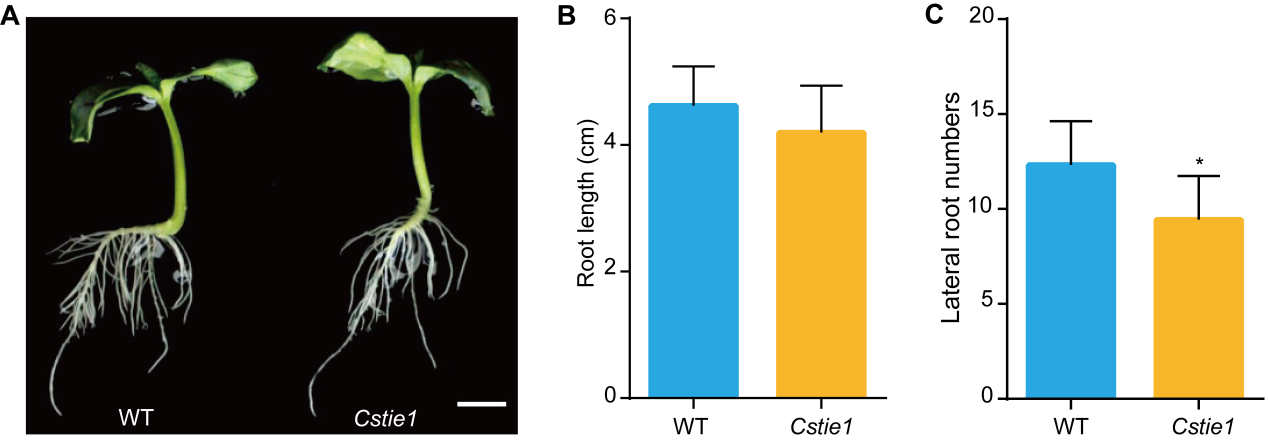


**Supplemental Figure S3 Root length analysis of *Cstie1* mutants. A** Representative images of roots of WT and *Cstie1* mutants 5-day-old seedlings. Scale bars, 1 cm. **B** and **C** Statistical analysis of root length (B) and lateral root number (C) of *Cstie1* mutants 5-day-old seedlings. Significance analysis was conducted with the two-tailed Student’s *t*-test (* *p* < 0.05). Values are means ± SD, n = 9.

**Table S1** Genes information used in this study.

| **Gene name** | **Species** | **Accession** |
| --- | --- | --- |
| *TIE1* | *Arabidopsis thaliana* | AT4G28840 |
| *TIE2* | *Arabidopsis thaliana* | AT2G20080 |
| *TIE3* | *Arabidopsis thaliana* | AT1G29010 |
| *TIE4* | *Arabidopsis thaliana* | AT2G34010 |
| *CsTIE1* | *Cucumis sativus* | CsaV3_2G002110 |
| *CsaV3_1G021830* | *Cucumis sativus* | CsaV3_1G021830 |
| *CsaV3**_4G008420* | *Cucumis sativus* | *CsaV3_4G008420* |
| *CsaV3_4G033280* | *Cucumis sativus* | CsaV3_4G033280 |
| *CsAGL16* | *Cucumis sativus* | CsaV3_3G048150 |
| *CsCYP707A4* | *Cucumis sativus* | CsaV3**_**4G034320 |
| *CsUBI* | *Cucumis sativus* | CsaV3_5G031430 |

**Table S2** Primers used in this study.

| **Primers for qRT-PCR** | |
| --- | --- |
| *CsAGL16-F* | GGAGCCCACATAAGCAGTATT |
| *CsAGL16-R* | TCTGGTGCCTCATTGTCTTG |
| *CsCYP707A4*  *CsCYP707A4*  *CsTIE1-F* | CAGACAAGGAACATGCCAATC  CATCAGCTACAGCCTCTCTAAA  TGGGCTGTGCTGCTTATT |
| *CsTIE1-R* | CGATTCATGAGTTGAGGAGTAAGA |
| *CsUBI-F* | CACCAAGCCCAAGAAGATC |
| *CsUBI-R* | TAAACCTAATCACCACCAGC |
| **Primers for genetic transformation and gene amplification** | |
| *CsTIE1-BsF* | ATATATGGTCTCGATTGCTTCATCTCCTAATTCCTAGTT |
| *CsTIE1-F0* | TGCTTCATCTCCTAATTCCTAGTTTTAGAGCTAGAAATAGC |
| *CsTIE1-R0* | AACCAACTCCCAACCCTCTCTGCAATCTCTTAGTCGACTCTAC |
| *CsTIE1-BsR* | ATTATTGGTCTCGAAACCAACTCCCAACCCTCTCTGCAA |
| *CsTIE1-clone-F* | ATGGGGAGTGGTTATTTTGG |
| *CsTIE1-clone-R* | TGTTAGTTGAGAATCACCATATC |
| **Primers for dual-luciferase reporter analysis** | |
| *CsAGL16-62-SK-F* | GCTCTAGAACTAGTGGATCCATGGGGAGAGGCAAAATTGTAA |
| *CsAGL16-62-SK-R* | TCGACGGTATCGATAAGCTTCTAGCGTAGTTGTAGTCTGCCC |
| *CsTIE1-62-SK-F* | GCTCTAGAACTAGTGGATCCATGGGGAGTGGTTATTTTGG |
| *CsTIE1-62-SK-R* | TCGACGGTATCGATAAGCTTTTAGATTGATAATCTCAACTCCAAA |
| *CsCYP707A4-0800 -F* | TCGACGGTATCGATAAGCTTGATGATAGGATAATGTTATTTT |
| *Cs CYP707A4-0800 -R* | GCTCTAGAACTAGTGGATCCCACACAAGGACATCCAAGTATA |
| **Primers for Co-IP analysis, firefly luciferase complementation imaging (LCI) assay** | |
| *CsTIE1-CoIP-F* | TCTGCAGGGGCCCGGGGTCGACATGGGGAGTGGTTATTTTGGGGAGA |
| *CsTIE1-CoIP-R*  *CsAGL16-cLUC-F*  *CsAGL16-cLUC-R* | CCTCGCCCTTGCTCACCATGGTACCGATTGATAATCTCAACTCCAAATCT  GTACGCGTCCCGGGGCGGTACCATGGGGAGAGGCAAAATTG  TTGTAGTCCATTTGTTGGATCCCTAGCGTAGTTGTAGTCTGCCC |
| *CsAGL16-nLUC-F* | GGGGGACGAGCTCGGTACCATGGGGAGAGGCAAAATTG |
| *CsAGL16-nLUC-R* | CGCGTACGAGATCTGGTCGACGCGTAGTTGTAGTCTGCCCAG |
| *CsTIE1-cLUC-F* | GTACGCGTCCCGGGGCGGTACCATGGGGAGTGGTTATTTTGGG |
| *CsTIE1-cLUC-R* | TTGTAGTCCATTTGTTGGATCCTTAGATTGATAATCTCAACTCCAAATCTAG |
| *CsTIE1-nLUC-F* | GGGGGACGAGCTCGGTACCATGGGGAGTGGTTATTTTGGG |
| *CsTIE1-nLUC-R* | CGCGTACGAGATCTGGTCGACGATTGATAATCTCAACTCCAAATCTAGC |
| **Primers for** **yeast two-hybrid analysis** | |
| *CsAGL16-AD-F* | GGAATTCATGGGGAGAGGCAAAATT |
| *CsAGL16-AD-R* | CGGGATCCCTAGCGTAGTTGTAGTCTGCC |
| *CsTIE1-BD-F* | TCCCCCCGGGATGGGGAGTGGTTATTTTGG |
| *CsTIE1-BD-R* | CGGGATCCTTAGATTGATAATCTCAACTCCAAA |
